# Ethnobotany of the Sierra Nevada del Cocuy-Güicán: climate change and conservation strategies in the Colombian Andes

**DOI:** 10.1186/s13002-018-0227-6

**Published:** 2018-05-05

**Authors:** Mireia Alcántara Rodríguez, Andrea Angueyra, Antoine M. Cleef, Tinde Van Andel

**Affiliations:** 1 Faculty of Archaeology, Leiden University, Einsteinweg 2, 2333 CC Leiden, The Netherlands; 2 Laboratorio de Botánica y Sistemática, Departamento de Ciencias Biológicas, Universidad de los Andes, Cra 1 No. 18A – 12, 111711 Bogotá, Colombia; 3 Institute for Biodiversity and Ecosystem Dynamics, University of Amsterdam, P.O. Box 94248, 1090 GE Amsterdam, The Netherlands; 4 Wageningen University, Biosystematics Group, Droevendaalsesteeg 1, 6708 PB Wageningen, The Netherlands; 5 Naturalis Biodiversity Center, PO Box 9517, 2300 RA Leiden, The Netherlands

**Keywords:** Alpine ecosystems, Sierra Nevada del Cocuy, Campesinos, Climate change, Colombia, Conservation, Local perceptions, Páramos, Useful plants

## Abstract

**Background:**

The *Sierra Nevada del Cocuy-Güicán* in the Colombian Andes is protected as a National Natural Park since 1977 because of its fragile páramo ecosystems, extraordinary biodiversity, high plant endemism, and function as water reservoir. The vegetation on this mountain is threatened by expanding agriculture, deforestation, tourism, and climate change. We present an ethnobotanical inventory among local farmer communities and discuss the effects of vegetation change on the availability of useful plants.

**Methods:**

We used 76 semi-structured, 4 in-depth interviews, and 247 botanical collections to record the ethnoflora of the farmers and surveyed from the high Andean forest to the super-páramo, including native and introduced species. We organized 3 participative workshops with local children, high school students, and campesinos’ women to share the data we acquired in the field and empower local plant conservation awareness.

**Results:**

We encountered 174 useful plants, most of them native to the area (68%) and almost one third introduced (32%). The Compositae was the most species-rich family, followed by Lamiaceae, Poaceae, and Rosaceae. The majority of plant species were used as medicine, followed by food, firewood, and domestic tools. Local farmers reported declining numbers of páramo species, which were now only found at higher altitudes than before. Although our informants were worried about the preservation of their natural resources and noticed the effects of climate change, for several commercial species, unsustainable land use and overharvesting seemed to be the direct cause of declining medicinal plant resources rather than climate change.

**Conclusions:**

We recommend conservation plans that include vegetation monitoring, people’s perceptions on climate change, and participative actions with the communities of the Sierra Nevada del Cocuy-Güicán.

**Electronic supplementary material:**

The online version of this article (10.1186/s13002-018-0227-6) contains supplementary material, which is available to authorized users.

## Background

Climate change affects altitudinal plant distribution in high-elevation tropical mountains [1–3]. Perceptions on climate change in mountain ecosystems indicate that local people can give relevant insights about climate change dynamics as they are narrowly acquainted with its surroundings [4, 5]. From an ethnobotanical approach, climate change affects human-vegetation dynamics, like altering the patterns of planting and harvesting in the Himalayas [5], disrupting traditional plant practices in British Columbia [6], and affecting the diversity of useful flora in alpine ecosystems, and therefore threatening the traditional knowledge associated with these plants [5, 7]. These studies stress the need to consider local people’s perspectives to reduce the impacts of climate warming. Changes in plant diversity as a consequence of climate processes show alarming effects on plant population over time [8–13]. Predictions on the effects of climate warming in the Andean ecosystems include displacement, adaptations (physiological changes), and local extinction of plant communities [8–10]. Ethnobotanical research in Andean mountain ecosystems have mostly focused on medicinal plant use by local communities [14–16]. Research on non-medicinal plants of importance for the inhabitants of high altitude zones, or on local perceptions on the decline of useful plants related to climate change are lacking. Apart from climate change, agriculture, pasture, and logging activities constitute the main drivers of deforestation of the Andean tropical forests and high altitude tropical wetlands, locally known as páramos [17–19].

In this paper, we present the results of an ethnobotanical inventory in the *Sierra Nevada del Cocuy-Güicán* (from now on “the SN Cocuy-Güicán”) and discuss the possible effects of climate and land use change on the future availability of useful plants for local farmer communities. The *SN Cocuy-Güicán* rises in the northern range of the Eastern Cordillera of the Colombian Andes. Since 1977, this region is protected within a National Natural Park (NNP-Cocuy) because of its fragile páramos, extraordinary biodiversity and endemism, and its function as a corridor for migratory species under conditions of climate change [20]. Páramos and Andean forests provide drinking water to Colombia’s large cities, like Bogotá, Medellín, and Bucaramanga. With the largest extension of glaciers in Colombia, the SN-Cocuy-Güicán is a valuable freshwater reservoir that supplies the Orinoco and the Magdalena River basins [21]. The local population consists of indigenous U’wa people, who occupy the eastern flanks of the SN Cocuy-Güicán, and more recent settlers (“colonos” or “campesinos”). The latter are farmers who inhabit the western flanks and were the focus group of our ethnobotanical research. The vascular flora of the páramo of SN Cocuy-Güicán is well documented [22–27], but the few ethnobotanical studies in this area focused on few medicinal plants [28], potatoes [29] or specific plant families such as Ericaceae [30]. Previous ethnobotanical research among farmers in the same Boyacá department (in Guacamayas at 32 km downslope from El Cocuy and in Villa de Leyva at 300 km distance) highlights the relevance of the useful flora for preserving their cultural heritage and biodiversity [31, 32].

Since the colonial period, human pressure on the páramo ecosystems increased due to social and economic changes [33]. New productive land systems replaced the more sustainable indigenous agricultural practices, based on altitudinal and seasonal zonation of land use. The introduction of exotic species (wheat, peas, broad beans) was combined with the application of European land use techniques (monocultures) and the introduction of cows and sheep in high altitude rural areas. The campesinos group emerged during the Spanish colonization as a consequence of settlement policies and the demand for farm labor, displacing the indigenous groups towards remote areas in the mountains [34].

In the SN Cocuy-Güicán, the ecological effects of the post-colonial land use changes are clearly visible. First native species were exploited like *Quercus humboldtii* and *Polylepis quadrijuga*. Later on, in deforested areas, there was a shift to introduced species like *Eucalyptus globulus* and *Pinus patula* to produce wood for construction, fuel, and paper production. European and North American grasses (e.g., *Anthoxanthum odoratum*, *Dactylis glomerata*, and *Lolium perenne*) and a tropical African grass (*Pennisetum clandestinum*) were introduced as fodder. Besides farming activities, tourism as a source of income increased considerable in the last decades [17]. Recent complaints by campesinos and indigenous groups about unsustainable and destructive practices by tourists led to the closure of the National Park from the February 2016 to April 2017, until new policies were introduced. The tropical highland ecosystem of the SN Cocuy-Güicán is vulnerable to climate change effects [21, 35–37]. The campaign to promote the area as an UNESCO World Heritage Site [38] demanded an inventory of useful plants. Although previous studies [28, 39] pointed out that local farmers’ plant knowledge is intertwined with the U’wa indigenous group, we documented the ethnoflora of the campesinos and tried to detect potential declining resources and their relation to climate change. We posed the following questions:

1. What are the plant species used by the campesinos?
2. At what altitudes do they collect useful plants?
3. What is the proportion of native versus introduced species?
4. Have the campesinos noticed a reduction in plant availability?
5. Could potentially declining plant resources be associated with climate change?

We expected that local farmers would use a large number of plants because they rely on their natural resources since the colonial era. We anticipated to find a greater proportion of native species than in nearby Andean areas [31, 32], as the SN Cocuy-Güicán is a quite isolated mountain with high levels of plant diversity [22–27]. We also expected a reduced availability of plant resources due to overharvesting practices. We hypothesized that farmers had to collect plants from higher altitudes than in the past as a consequence of climate change and vegetation zones moving upwards [11–13]. We hope that this study will provide international support to preserve the cultural heritage and the fragile páramos of the SN Cocuy-Güicán. Our inventory serves as a basis for implementing projects on environmental education and sustainable development, such as ethnobotanical field trails for children and tourists, and useful plant workshops in schools or community groups.

## Methods

### The study area

**Fig. 1:**
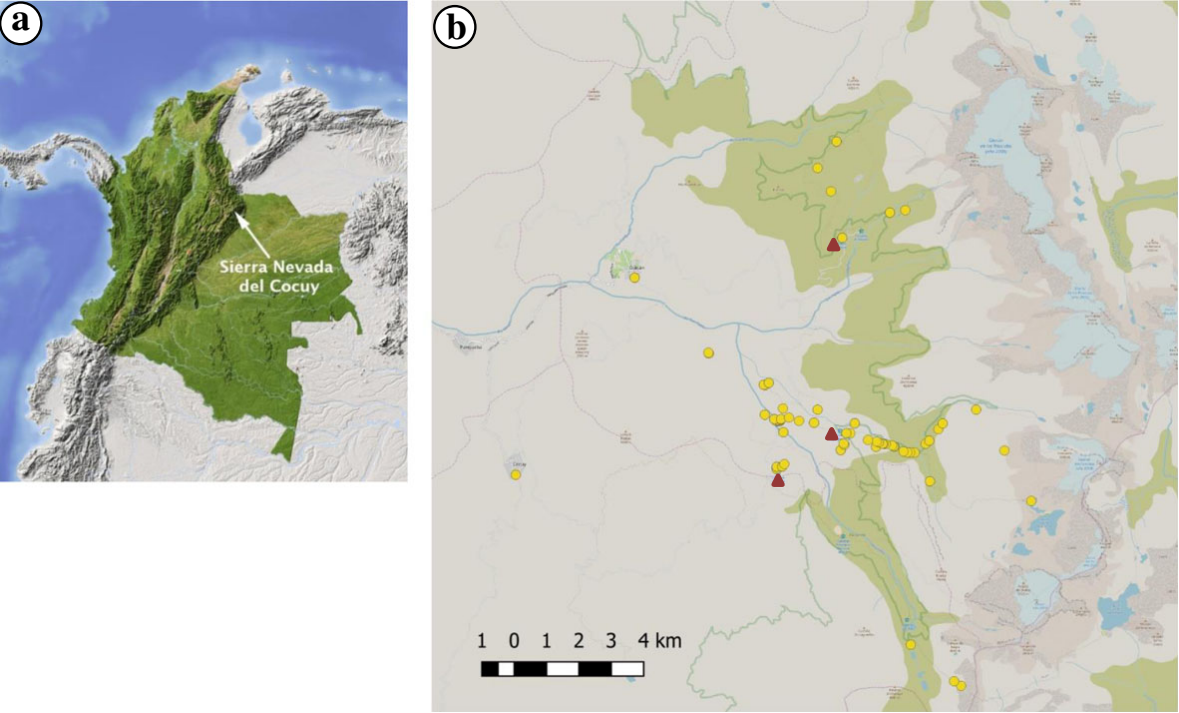
**a** Location of Sierra Nevada del Cocuy in the Andes mountain range [38]. **b** Map of the surveyed area and collection sites

Fieldwork formed part of a botanical expedition by the University of Amsterdam between January and March 2017 in the SN Cocuy-Güicán (Fig. 1), located in the highest summits of the Cordillera Oriental (between 6° 26′ 0″ N and 72° 17′ 0″ W) within the Departments Boyacá and Arauca. The natural vegetation on the west side of the Sierra Nevada ranges from xerophytic forest (600 m asl) to páramo in the alpine zone, with the highest peak in the Ritak’uwa Blanco (5380 m). The SN Cocuy-Güicán’s páramo ecosystem is divided in three vegetation zones: grass páramo (3900–4350 m), subpáramo (3500–3900 m), and superpáramo above 4350 to the snow cap [22, 24]. The high Andean forest corresponds in large part to a “paramisation” zone (3300 to 3500 m), where the forest is replaced by herbaceous páramo-like vegetation [17]. The Andean forest is located between about 2500 to 3300 m. Glacial lakes are numerous: the largest ones are the Laguna Grande de la Sierra (4450 m), La Plaza (4200 m), La Cuadrada (4042 m), and the Laguna Grande de los Verdes (3975 m). The climate is typical of páramo ecosystems: cold at night and warmer during daytime, frosting in the superpáramo zone, with influence of trade winds from the Atlantic and the Amazon basin [35]. Precipitation contrasts between the eastern and western slopes and no thermal seasonality occurs, except for the annual confluence of dry and rain season. The mean maximum temperature has increased 2 °C in 34 years (1976–2010) [21]. It is expected that the temperature will increase with an average of 3 °C (± 1.5 °C) by 2100 [36, 40].

The vascular flora of the páramo includes about 482 species and the superpáramo accounts for 175 species [23, 38]. The local population is distributed in two main municipalities: Güicán (pop. 5920) and El Cocuy (pop. 5383) [41]. We worked with farmers who inhabit the higher areas of the western flanks of the SN Cocuy-Güicán, within the Vereda de San Antonio de la Cueva (Vereda de la Cueva), Parada de Romero, and Lagunillas sectors (Fig. 1).

### Data collection

We walked into the field and along existing mountain trails with staff from the NNP-Cocuy and local farmers (one young girl of 9 years old and 9 men ca. 20 to 55 years old) to collect useful plant specimens from our base camps in La Esperanza, Kanwara, and Guaicaní (Fig. 1). Farmers pointed out useful plants to us, but we also selected plants ourselves to verify whether they were used. We also brought fresh and dried specimens to the campesinos’ villages to discuss the previously collected ethnobotanical information during home interviews (33 men ca. 20 to 75 years old and 35 women ca. 9 to 80 years old). We used these specimens in the interviews or we asked our collaborators to show us the plants they were mentioned. We combined 76 semi-structured interviews with 4 in-depth interviews and participant observation with local people, following the Code of Ethics of the International Society for Ethnobiology [42]. We used snowball sampling [43] to find collaborators who were willing to share their ethnobotanical knowledge and indicate well-known plant experts in the community. Valid and reliable data was assured by interviewing a high number of participants and selecting knowledgeable plant people. We focused on plants known and used by the campesinos, either in the past or the present, because their general floristic knowledge would allow us to detect plant use changes that could be related to potential declining plant resources or new regulations by the park authorities. Interviews were formulated around the following questions: What plants do campesinos use and for what purposes (including plant part, preparation, application)? Where these plants are collected (habitat, altitude) and what is their domestication status (cultivated, wild)? Have you noticed any change in the use or availability of these plants? What is the cause of declining resources of useful plants? From whom did you learn how to use these plants? All interviews were held and recorded in Spanish.

Our inventory covered all ecosystems: Andean forest, sub-páramo, páramo, and super-páramo within the NNP-Cocuy. We collected three duplicates of each specimen and we recorded their uses, local names, habitat, habit, and location, using a high-sensitivity GPS device (Etrex-GARMIN). We surveyed in the markets of Cocuy and Güicán to document the commercialization of (medicinal) plants from our study area. Vouchers were deposited at the herbaria of the National University of Colombia (COL) and the Andes University (ANDES) in Bogota. The specimens were identified at the COL herbarium.

### Data analysis

We organized our data in an Excel table (Additional file 1) with the following information: collector, collection number, collection date, geographical coordinates, family, scientific name, specialist who identified the specimen, plant description, collection locality, domestication status, native or introduced, habit, habitat, vernacular names, language, uses (parts used, preparation, application or illnesses treated), and name of the informants. We consulted the *Catálogo de Plantas de Colombia* [44] to know whether a plant was native, endemic, or introduced and we checked the plant list [45] for the current scientific names according to the APG III system. We categorized plant uses in medicinal (including veterinary uses), food (including seasonings), wood (including firewood, construction, fences, and tools), domestic (including handicrafts and decorative items), fodder, ritual, dye, and restoration. The latter category included those species that were used to restore disturbed páramo ecosystem. We also asked for the campesinos’ perspectives and personal experiences related to climate change and plant availability.

### Sharing data with the community

**Fig. 2:**
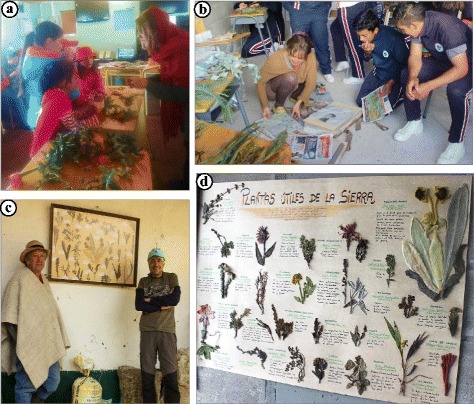
**a** Workshop with children of the *Vereda de la Cueva*. Girls are choosing the plants to make an infusion according to their knowledge. *Passiflora mixta* vine on the table. **b** La Normal students pressing plants with AA. **c** One of the poster-herbarium left in *La Esperanza* farmhouse*.* **d** Detail of the other herbarium with plant uses, scientific and common names

Ethnobotanical studies are based on active participation of the local population. Therefore, ethnobotanists should compensate local people for their collaboration, taking into account their needs to promote conservation projects and improve the community well-being [31, 46–49]. On the request from one of our local collaborators, we organized three dynamic workshops towards the end of our fieldwork, during which we used fresh plants, a poster with 20 dried native specimens glued on it, and our own field-collected material to explain ethnobotanical concepts and methods to the farmer communities. The workshops were entitled “What is the use of this plant? Useful plants of the SN Cocuy-Güicán: sharing the ancestral knowledge of its inhabitants.” The workshops allowed us to share the ethnobotanical data acquired through this research with the community and highlight the value of their botanical heritage to less knowledgeable community members (youth, migrants). Workshops were directed to all children (varying in age from ca. 9 years old, 20 children, 4 girls, 16 boys) from three primary schools of the *Vereda de la Cueva* (Fig. 2), women (ca. 20 to 70 years old, 9 women) from the association of Luz Dari in the *Vereda de la Cueva* and teenagers (ca. 16 to 17 years old, 30 students, ca. 50% of each gender) from the *La Normal* school in Güicán town (Fig. 2). We focused on these groups to increase social diversity and inclusion, as we worked mainly with adult men during our field surveys. During the activities, we shared the preliminary results of our field research, so local participants benefited from the exchange of knowledge and we also gathered new information on plants for which our data were unclear. We also encouraged the participants to be “Ethnobotanists for a day”, applying our methodology: pressing specimens, asking their partners about plant information, and labelling the vouchers with uses and names. In addition, we discussed about local concerns regarding declining plant resources and changes in traditional plant use.

## Results

**Fig. 3:**
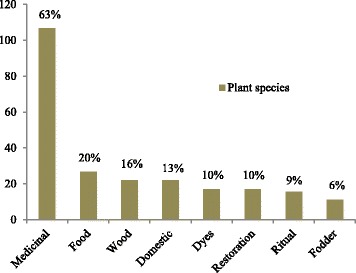
Useful plant categories. Legend: proportion of plant species by use categories

We conducted 76 interviews (35 women, 41 men) and collected 149 specimens, including 143 vascular plants, three lichens, two bryophytes, and one mushroom. In total, we recorded 174 useful species, grouped in 126 genera and 59 families, which corresponded to 178 vernacular names. Scientific names had mostly Greek and Latin roots, but at least 23 names were derived from the Amerindian Muisca and Laches’ languages (ancestors of the U’wa group), belonging to the Macrochibcha linguistic family [33, 34, 50, 51]. We identified 168 plants at species level and six at genus level (Additional file 1). The most species-rich family was Compositae (30 spp.) followed by Lamiaceae (12), Poaceae (10), Rosaceae (9), Apiaceae (8), Solanaceae (7), and Leguminosae (6). Most species (108 spp.) had medicinal uses, followed by species used for food, wood and domestic uses. Similar percentages were observed for dye plants, restoration, and ritual plants, while fewer species are used as fodder (Fig. 3).

The most cited health conditions for which people used medicinal plants were colds (flu, sore throat, cough), wounds, stomachache, heart problems, blood circulation, and female reproductive health issues (menstruation cramps, abortion, child delivery, vaginal baths). Although edible plants were numerous (36 spp.), some constituted famine or emergency foods, used in times of scarcity or special energy demands [52, 53]. For example, the raw marrow of *Espeletia* stem rosettes was eaten during the amine period caused by the violent conflict between the Conservative and Liberal parties in the 1950s. Other plants were consumed fresh to obtain energy and alleviate the thirst during high physical demand activities in the mountains, such as the leaves of *Echeveria bicolor*, bulbs of *Cyrtochilum revolutum*, the entire lichens of *Thamnolia vermicularis*, and fruits of *Vaccinium floribundum*. Potatoes (*Solanum tuberosum*) and long onions (*Allium fistulosum*) were the most popular crops cultivated in the SN Cocuy-Güicán, both grown for home-consumption and the market. Lesser crops were peas (*Pisum sativum*) and broad beans (*Vicia faba*), but these were sometimes also purchased in the markets. Maize and wheat were cultivated for subsistence in a few plots.

**Fig. 4:**
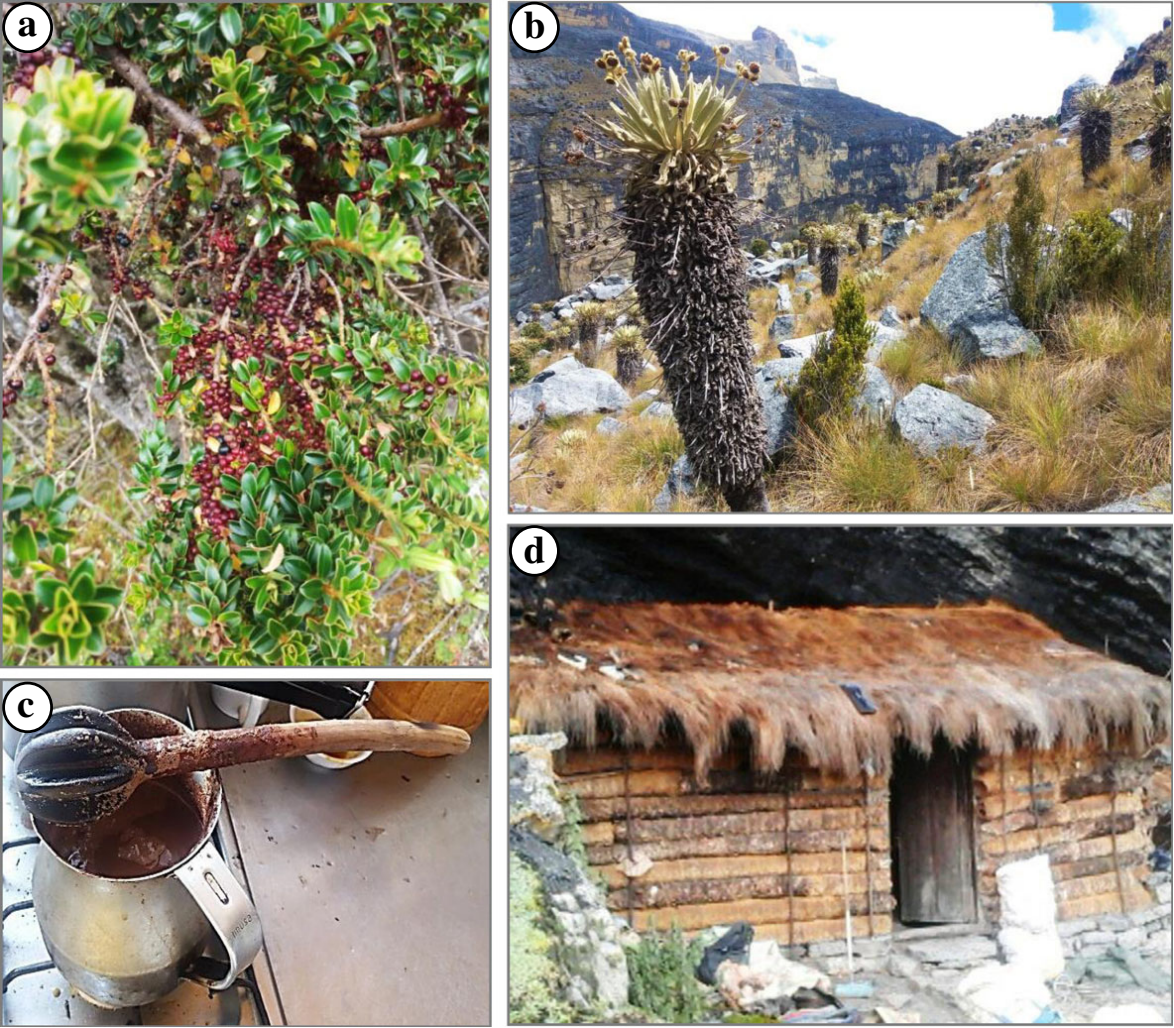
**a**, **c** *Myrsine dependens* shrub, detail of the fruits and branches; spoon made from *M. dependens* wood. **b**, **d** *Espeletia lopezii*. Páramo’s house made from *Espeletia* (walls), *Calamagrostis effuse* (roof) and *Pinus patula* (door)

Within the wood category, most of the plants were used to obtain firewood (19 spp.), followed by species to make fences (11), house construction (8), and tools (5). The wood of *Myrsine dependens*, known as “cucharo,” was used to make spoons to stir hot chocolate (Fig. 4). Currently, the manufacture of wooden spoons is nearly abandoned, just like the thatching of roofs with *Calamagrostis effusa* (Fig. 4), *Agrostis boyacensis*, or *Cyperus luzulae.* Other lost plant practices include the building of walls with *Espeletia* spp. (Fig. 4) and the making of beds with the lily *Orthrosanthus chimboracensis.*

Domestic uses were mentioned for 22 species, but local inhabitants said its relevance had decreased, just like the use of plants for natural dyes and rituals. *Hypericum juniperum* and *H. laricifolium* were used as brooms to clean the house before plastic brooms appeared in the SN Cocuy-Güicán. *Crescentia cujete* and *Lagenaria siceraria* were used to store and carry “chicha,” an ancestral indigenous drink made from fermented corn. Chicha is no longer made by the campesinos as it has been replaced by the commercial beer and other alcoholic beverages. Globalization has influenced the farmer’s previous self-sufficiency on natural resources and promoted modern lifestyles with less reliance on the local vegetation and gardens and a greater dependence on industrial products.

We also encountered “new” plant uses that emerged in 1998, during the restoration of degraded páramo vegetation. Some of the species that the NNP-Cocuy employees used in their nursery garden “El Alto de la Cueva” at 3950 m were *Buddleja bullata*, *Calamagrostis effuse*, *Diplostephium* spp., *Draba cocuyensis*, *Espeletia* spp., *Lupinus pubescens*, *Myrsine dependens*, *M. guianensis*, *Pentacalia corymbosa*, *Polylepis quadrijuga*, *Senecio niveoaureus*, *S. wedglacialis*, *Vallea stipularis*, *Viburnum hallii*, and *Weinmannia microphylla*. Most of these were traditionally used by the campesinos (see Additional file 1) but are now used to restore páramo areas that have been degraded by the cattle or deforestation. The NNP-Cocuy employees used only native plants for restoration projects in the National Park.

**Fig. 5:**
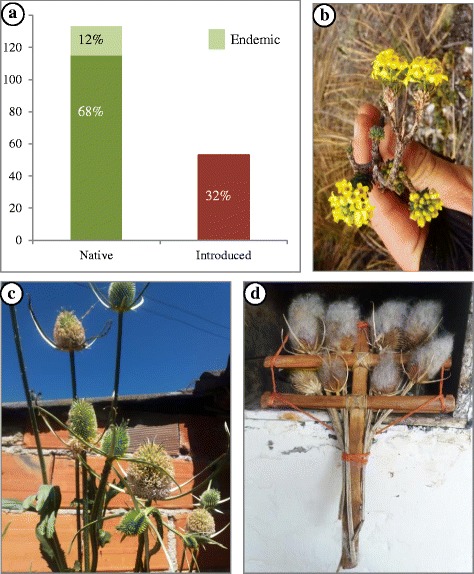
**a** Proportion of native, endemism, and introduced plants used by farmers. **b** *Draba litamo*, an endemism of the Sierra Nevada del Cocuy-Güicán. **c** *Dipsacus fullonum*, an European introduced herb. **d** Domestic tool made from the inflorescences of *D. fullonum* to clean the *ruanas*

**Fig. 6:**
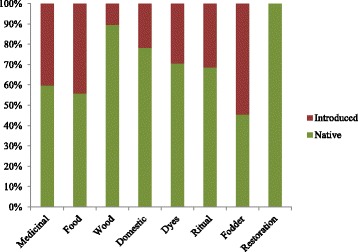
Proportion of introduced versus native plants by use categories

Most plants (68%) reported by our informants were of native origin (Fig. 5), with almost 12% of the species endemic to the Eastern Cordillera (e.g., *Draba litamo* (Fig. 5), *Gynoxys paramuna*, *Halenia gentianoides*, *Pentacalia guicanensis*). Most of the introduced useful plants were originally from Europe, like *Dipsacus fullonum* (Fig. 5), of which the spiny inflorescences are used by farmers to clean their “ruanas,” the typical Boyaca ponchos made from sheep wool. We found more native plants in the categories wood (90%), dyes (70%), rituals (69%), and domestic tools (78%) than in those used as medicine (60%), food (56%), and fodder (46%) (Fig. 6). Although most useful plants (66%) were native to the páramo, some local people stated that it was easier to cultivate medicinal plants in their home yards (mostly introduced species) than climbing up the mountain to collect species from the wild.

When the settlers brought livestock to the Andean páramos, many exotic plants from Europe were introduced as fodder. Examples are *Dactylis glomerata*, *Pennisetum clandestinum*, and *Lolium perenne*. The colonization and expansion of these grasses have been stimulated with the increase of grazing areas, created by clearing and burning patches of páramo vegetation and cutting large areas of Andean forest, causing paramisation. Páramo species quickly pioneering arrive first and with them also native weedy species (e.g., *Paspalum bonplandianum*, *Lachemilla orbiculata*, and species of *Juncus*, which are rare in pristine páramo. Also other exotic species arrived with them such as *Hypochaeris radicata*, *Digitalis purpurea*, *Rumex acetosella* (pioneering after potato harvest), *R. obtusifolius*, and *Trifolium pretense.*

### Market survey

We identified 11 medicinal and edible species harvested from the NNP-Cocuy in the markets of Cocuy and Güicán: *Aloe vera*, *Aloysia citriodora Alternanthera lanceolata*, *Citrus x aurantium, Coriandrum sativum*, *Cymbopogon citratus*, *Matricaria chamomilla*, *Melissa officinalis*, *Ocimum basilicum*, *Petroselinum crispum*, and *Senna occidentalis*. Except for *A. lanceolata* and *S. occidentalis*, all species were cultivated exotics and sold in herbal corsages. Our informants stated that in the past, a greater diversity of native plants was sold at the markets, including *Draba litamo* and *Niphogeton dissecta*. The current scarcity of these species, as perceived by the campesinos and observed during our field walks, and the restrictive policies by the NNP-Cocuy on harvesting páramo flora could explain these changes in plant diversity on the market.

### Climbing higher to harvest useful plants

The farmers we interviewed mentioned a reduction of native and especially medicinal plant resources. Species like *Draba litamo*, *Senecio wedglacialis*, *Niphogeton dissecta*, *Lepidium bipinnatifidum*, *Passiflora mixta*, and *Acaena elongata* were more difficult to find and had shifted to higher altitudes. These changes could be caused by climate change, as the temperatures have increased 2 °C in the SN Cocuy-Güicán in less than four decades [21] and the páramo vegetation tends to colonize areas above their distribution range in response of climate warming [11–13]. Farmers reported that the weather was already unpredictable and the decrease of the snow causing water shortage affected the vegetation, including their crops. They indicated that crops like maize and wheat were now cultivated at higher altitudes. One farmer reported: “50 years ago, some cultivars were planted in a specific time of the year, nowadays they can be planted all year because the climate has changed.” Another farmer commented: “This [climate change] can be good in some sense, for those who have water to maintain them, but we cannot predict the weather anymore.”

Local inhabitants had noticed the retreat of the glaciers and the decrease of snow on the mountain peaks. Many farmers commented on this phenomenon, with statements like “The most affected by climate change are the glaciers, the decrease in water” and “If there is less water, some plants will be affected, especially those that live in the marshes” and “Plants are now growing where there was snow before”. Most farmers, however, related the decrease of the snow to tourist activities, such as stepping and littering on the snow. The farmers that were previously informed by the NNP-Cocuy campaigns related it to climate change. As the snow continues to disappear, the páramos’ freshwater supply will decrease, which will directly affect the farmers subsistence. As observed by local communities in the Himalayas [5] and British Columbia [6], planting and harvesting patterns are changing, and therefore the behavior and farming habits of their inhabitants must adapt, in a relatively short time, to these changes.

The campesinos we interviewed mentioned that they perceived an increasing scarcity of *Draba litamo*, known as “lítamo real”, a small herb that is made into an infusion or macerated in wine to revitalize and obtain eternal youth. For several days, we surveyed field areas that farmers pointed out as the (former) natural habitat of *D. litamo*, but we could only find a few specimens at the very end of our fieldwork. Some of the medicinal species were previously gathered in large amounts to be sold at the market, like the *D. litamo*. The increasing scarcity or absence of this plant indicated dwindling resources of this species. In this case, however, overharvesting seemed a more important factor causing the decline in medicinal plant resources than climate change.

The decline in other medicinal plants that are now found in higher altitudes could be affected by overharvesting practices rather than climate change due to its commercial and therapeutic value. Nevertheless, climate change processes could be playing a role in their ecological distribution and abundance that might be observed in long term.

### Community workshops

**Fig. 7:**
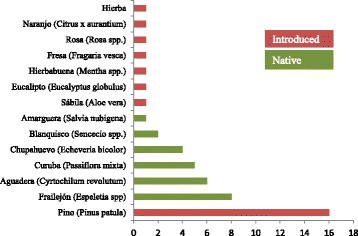
Proportion of the most popular plants for the children of the *Vereda de San Antonio de la Cueva* schools

During the three workshops, we observed great enthusiasm among all groups and high participation rates when exchanging plant knowledge, which could reinforce the community bonds [46, 49]. We left two posters with 20 dried plant specimens in the *Esperanza* farmhouse (Fig. 2) and the *Capilla* social building. Such “herbarium posters” visualized useful native plants and created awareness of the diversity and richness of the local floristic resources, which can help to increase conservation efforts by local people and tourists. During the workshop with the primary schools of our study site, we asked all children (20) to draw and write the names of three plants they considered most important to them. The pine tree (*Pinus patula*) emerged as the most popular plant, followed by the “frailejón” (*Espeletia* spp.), the “aguadera” orchid (*Cyrtochilum revolutum*), the “curuba” vine (*Passiflora mixta*), and the succulent “chupahuevo” (*Echeveria bicolor*) (Fig. 7). The numerous citations of páramo plants showed that children were connected to their high altitude environment and that retention of ancestral plant knowledge was present to a certain degree, although most of them considered an introduced tree (*P. patula*) as the most relevant one, probably because of its abundance in their environment.

## Discussion

### Changes in plant uses

As concluded from the campesinos’ information on useful plants, the traditional uses of at least 18 species had declined over time or disappeared altogether. However, the farmers we interviewed still had considerable knowledge on useful plant resources in the SNN-Cocuy, as they pointed out 174 useful species. The skepticism of our informants about modern medicine, its price, and the lack of medical facilities in the area could be an explanation for the popularity of natural plant medicine. Edible plants were numerous (although many of them exotic), but emergency food was seen as a great ally in the extreme conditions of the high mountains. Farmers had noticed a decline in the volume and diversity of domesticated edible crops over time. The land became less fertile due to the use of agro-chemicals and overexploitation [54]. The lack of incentives in agriculture, the high rural to urban migration rates in the last decades, and the decrease in the number of children per household (more sustainable but less workforce) have caused campesinos to shift to farming activities that include more cattle heads and less crops [54]. This less risky investment provides more meat-derived products to commercialize. The transition from a diverse and self-sufficient agriculture to monocultures of potatoes, maize, long onions, and intensive grazing has also been observed among farmers in nearby areas [31, 32] with the consequent loss of traditional agricultural knowledge.

The campesinos in the SN Cocuy-Güicán retained substantial knowledge about plants used for their wood, but in contrast with the local inhabitants of Villa de Leyva [32], the demand in this category was much lower, probably because the collection of native woody species for construction, fencing, or firewood had been restricted in the páramos by the park authorities. As logging activities in the park were prohibited, plastic, metal, and other building materials were now more popular than before. The restrictive policies in the páramo and superpáramo emerged from the unsustainable exploitation of some plant resources (e.g., *Espeletia spp.* and *Polylepis quadrijuga*), which were used for firewood, to make fences or to build houses (Fig. 4). The consequence of this large-scale extraction in the past, especially during the ninetieth and twentieth century [33] and the legal prohibitions afterwards, is that many traditional plant uses have been abandoned. The same native woody species, however, were now used for restoration projects by the employees of the NNP-Cocuy.

### Introduced plants

Comparable to other studies on Andean ethnobotany [31, 32], the proportion of native plants used by our study group (68%) was more than twice the number of introduced species. However, the farmers in the SNN-Cocuy used a considerable proportion of introduced plants to feed their cattle (55%), for consumption (44%), and to heal themselves (40%). Plantations of introduced trees like *Pinus patula* and *Eucalyptus globulus* have displaced and decreased the natural resources of native useful plants [55, 56]. Pine plantations cause soil acidification and water loss, which affects the original vegetation [57, 58]. However, introduced species also have become important for the subsistence of local farmers in the provision of food, medicine, and fodder. Although the discontinued use of native species may erode the traditional plant knowledge [32], the input of introduced plant medicine can also enrich the local pharmacopeia [59]. This was also observed in our study area, where many introduced medicinal species (*Mentha* spp.*, Origanum* spp., *Calendula officinalis*, *Borago officinalis*) were cultivated in house yards or purchased at the market, so their presence may have a limited impact on the natural environment.

In contrast to the indigenous community in the South Colombian páramo La Ortiga [15], most of the useful native plants (97%, including endemic species) in our study were found in páramo areas (from 3500 m). Only 3% of the useful native plants were found below this distribution range while 77% where found in the high Andean forest range (3200–4000) in La Ortiga [15]. Unlike in the neighboring municipality of Guacamayas [31], most of the useful plants in the SN Cocuy-Güicán were collected from the wild (59%), some were cultivated (28%), and few were gathered from both wild and cultivated sources (12%). All cultivated plants were introduced species, while plants that were both wild and cultivated were either naturalized exotics (fodder species like *Dactylis glomerata* or *Trifolium pratense*) or native species used for restoration by the NNP-Cocuy staff. These percentages of native versus introduced species were almost equal to the values found in Villa de Leyva [32] for the total of useful plants. By contrast, the proportion of native medicinal plants in the SN Cocuy-Güicán was a little higher than in Guacamayas [31] (Table 1).

**Table 1:** Proportion of native and introduced plants for three areas of study in Boyacá

Area of study	Medicinal plants	% Nat	% Introd	Total plants	% Nat	% Introd
Sierra Nevada Cocuy-Güicán	107	60	40	174	68	32
Villa de Leyva [32]	71	66	34	210	69	31
Guacamayas [31]	229	53	47			

*Nat* native, *Introd* introduced

## Conclusions

Unsustainable land use, including overharvesting practices, combined with the effects of climate change affect the natural population of useful plants in the páramos of the west slope of the SNN-Cocuy. Monitoring the spatial distribution of vegetation over time is required to obtain quantitative data on the decline of plant resources and the direct causes for this decline in order to apply the proper conservation policies. The Colombian Páramo Vegetation Database (CPVD) [60], the GLORIA-Andes database [61], and the almost 200 records of the Cocuy páramo vegetation (1972–2017) collected by Cleef [23, 38] could be used as sources to study floristic distributions in the past, register current alterations, and predict vegetation changes in the future. This study confirms the concern among local farmers about the melting snow, so it is crucial to include people’s perceptions on climate change to design effective conservation policies [4–6, 62–64]. During our workshops, we noticed that local farmers worried about the preservation of their natural resources. Local concerns can be solved with the implementation of environmental policies and active participation that take into account the local population needs [5, 49, 64, 65]. Courses on environmental conservation for local farmers are highly relevant, especially for those who are directly involved in the tourist business. Employees from the NNP-Cocuy, specialists on plant resources management and local people should work together to develop conservational strategies towards sustainable tourism and practices and accomplish the policies that were implemented since the opening of the NNP-Cocuy, such as obligatory-guided heritage tours, limited number of tourists, and no garbage disposal in the environment [66]. Placing local people as key actors will help to prevent the degradation of the floristic páramo resources and their cultural plant legacy.

### Suggestions for further research

The SN Cocuy-Güicán is the ancestral land of Muiscas and Laches, forefathers of the indigenous U’wa group [33]. Anthropologists have studied the U’wa’s land use and cosmovision [67–70], but a systematic study of their useful flora does not exist. Ethnobotanical research among the U’wa is essential to complete the inventory of useful plants of the SN Cocuy-Güicán. Through comparative analysis between the ethnobotany of farmers and the U’wa, it can become clear to which extent exchange of knowledge on plant use have taken place among these two groups that once lived surrounded by the same natural resources.

## Additional file

Additional file 1: Plant uses, scientific nomenclature, vernacular names and other ecological data of the flora used by the campesinos of the Sierra Nevada del Cocuy-Güicán in the Colombian Andes. (XLSX 56 kb)

## Abbreviations

NNP-Cocuy: National Natural Park del Cocuy
SN Cocuy-Güicán: Sierra Nevada del Cocuy-Güicán
